# Redox-Responsive Disulfide Bond-Bridged mPEG-PBLA Prodrug Micelles for Enhanced Paclitaxel Biosafety and Antitumor Efficacy

**DOI:** 10.3389/fonc.2019.00823

**Published:** 2019-08-27

**Authors:** Sheng Chang, Yanfei Wang, Tianyi Zhang, Xiaohui Pu, Lanlan Zong, Heyun Zhu, Luling Zhao, Bo Feng

**Affiliations:** ^1^College of Pharmacy, Jilin Medical University, Jilin, China; ^2^School of Pharmacy, Institute of Materia Medica, Henan University, Kaifeng, China

**Keywords:** redox responsion, mPEG-PBLA, prodrug micelles, paclitaxel, antitumor efficacy

## Abstract

The toxicity and side effects of traditional chemotherapeutic drugs are the main causes of chemotherapy failure. To improve the specificity and selectivity of chemotherapeutic drugs for tumor cells, a novel redox-sensitive polymer prodrug, polyethylene glycol-poly (β-benzyl-L-aspartate) (PEG-PBLA)-SS-paclitaxel (PPSP), was designed and synthesized in this study. The PPSP micelle was manufactured via high-speed dispersion stirring and dialysis. The particle size and zeta potential of this prodrug micelle were 63.77 ± 0.91 nm and −25.8 ± 3.24 mV, respectively. The micelles were uniformly distributed and presented a spherical morphology under a transmission electron microscope. In the tumor physiological environment, the particle size of the PPSP micelles and the release rate of paclitaxel (PTX) were significantly increased compared with those of mPEG-PBLA-CC-PTX (PPCP) micelles, reflecting the excellent redox-sensitive activity of the PPSP micelles. The inhibitory effect of PPSP on HepG2, MCF-7 and HL-7702 cell proliferation was investigated with MTT assays, and the results demonstrated that PPSP is superior to PTX with respect to the inhibition of two tumor cell types at different experimental concentration. Simultaneously PPSP has lower toxicity against HL-7702 cells then PTX and PPCP. Moreover, the blank micelle from mPEG-PBLA showed no obvious toxicity to the two tumor cells at different experimental concentrations. In summary, the redox-sensitive PPSP micelle significantly improved the biosafety and the anti-tumor activity of PTX.

## Introduction

Cancer is a major killer that endangers human life and health. Its mortality rate is much higher than that of AIDS, tuberculosis and malaria combined (1). Aside from surgery, chemotherapy is the most important therapeutic approach for cancer treatment (2). However, most chemotherapeutic drugs suffer from intrinsic limitations, such as poor water solubility, low bioavailability, poor selective distribution, rapid blood clearance (3, 4), serious side effects (5), and drug resistance of cancer cells as well (6, 7).

A drug delivery system (DDS) can overcome the above disadvantages of chemotherapy to a certain extent (8–12). Therefore, DDS research and development for cancer treatment is valued by academic institutions and industry. Over the past few decades, nanocarrier drug delivery systems (NDDSs) have played a vital role in improving the treatment effect of traditional chemotherapeutic drugs and reducing their side effects and toxicity (13). Polymer prodrugs have shown great potential in NDDSs because they can control drug release and minimize premature release from the blood circulation to the tumor cell matrix, eventually transporting drugs to the target cancer cells (14–17). However, traditional nanocarrier-mediated delivery systems usually exhibit sustained release behavior in the body, resulting in slow release of drugs from days to weeks (18). Furthermore, the drug cannot reach treatment concentrations in tumor tissue because of low drug loading and insufficient drug release, impairing the chemotherapeutic efficacy of the drug (19, 20). Therefore, effective treatment of cancer requires not only transportation of a sufficient amount of drug to the target tumor tissue but also rapid release of drug in tumor tissue to reach effective therapeutic concentration. Environment-responsive nano-micelle prodrugs are a new type of nanomedicine, without the disadvantages of the traditional NDDSs mentioned above. Compared with traditional NDDSs, drug loading is much higher and preparation is easier (21–27).

In recent years, with improved in-depth understanding of the development and formation of tumors, it has been found that the tumor microenvironment is very different from that in normal tissues, exhibiting a low pH and high levels of reduced glutathione (GSH) (28). Notably, the intracellular GSH concentration in tumor cells is ~100–1,000 times higher than the extracellular concentration (29–31) and several times higher than in corresponding normal cells (32). This high GSH level has been widely used in designing reduction-sensitive nanosystems, and disulfide bonds are widely used as a reduction-responsive linkage to facilitate rapid and differential release of chemotherapeutic drugs in tumor cells and to enhance antitumor activity (32, 33). Therefore, a polyethylene glycol-poly(β-benzyl-L-aspartate) (PEG-PBLA)- SS-paclitaxel polymer prodrug with disulfide bond linkages was designed as a drug delivery carrier to promote rapid and differential release of drug in cancer cells from the hydrophobic segment of the prodrug and to increase drug delivery in cancer cells to enhance anti-tumor activity.

Paclitaxel (PTX) is one of the most potent and successful chemotherapeutic drugs and has been widely applied in chemotherapy for treatment of a variety of cancers, such as breast, ovarian, non-small cell lung, colon, bladder, and head and neck cancer (34–37). However, the chemotherapeutic efficacy is far from satisfaction owing to its intrinsic physicochemical properties. PTX is classified according to the biopharmaceutics drug disposition classification System (BDDCS) as a class II drug, which has low solubility and extensive metabolism (38). Additionally, its lack of selectivity and cytotoxic effects in normal cells leads to serious and toxic side effects, such as hypersensitivity and cardiovascular toxicity (39, 40). To improve the therapeutic effect of paclitaxel in the present study, we describe a new type of PTX self-assembled prodrug, mPEG-PBLA-SS-PTX (PPSP), with disulfide linkages and reduction- responsive release properties, and its molecular structure was characterized by nuclear magnetic spectrogram. PPSP self-assembles to form homogeneous nanoparticles. The disulfide bond was used as a reduction-responsive linkage to facilitate rapid release of paclitaxel in tumor cells. PPSP micelles were prepared by combining high-speed dispersion stirring and dialysis methods. The physicochemical properties, reduction sensitivity, release behavior and anticancer activity of PPSP were evaluated and compared with those of PTX and mPEG-PBLA-CC-PTX (PPCP). To the best of our knowledge, this is the first attempt to develop an mPEG-PLBA-based disulfide bond-bridged micelle for PTX delivery.

## Materials and Methods

### Materials

Paclitaxel was purchased from Nanjing Jingzhu Bio-technology Co., Ltd. (Nanjing, China). Pyrene (purity 97%) was purchased from Shanghai Jingchun Bio-technology Co., Ltd. (Shanghai, China). Dialysis tube (MWCO 2000/350) was obtained from Shanghai Lvniao Technologies, Inc. (Shanghai, China). Glutathione (GSH), 4-(dimethylamino-pyridin (DMAP, purity > 98%), 3, 3′-Dithiodipropionic acid (DTDP, purity > 98%), Acetyl chloride (purity > 98%), CaH_2_ (purity > 98.5%) and *N, N*'-Dicyclohexyl-carbodiimide (DCC, purity > 98%) were purchased from Aladdin Reagent Co., Ltd. (Beijing, China). Succinic anhydride (purity > 99% GC), Acetonitrile hyper grade for HPLC, DMSO-d_6_, and CDCl_3_ were purchased from J & K Scientific Ltd. (Beijing, China). 3-(4,5-Dimethylthiazol-2-yl)-2,5-diphenyl Tetrazolium bromide (MTT, 5 mg/mL Sigma-Aldrich), Dulbecco's modified Eagle medium (DMEM), penicillin-streptomycin (Amresco, USA), 0.25% (w/v) trypsin-EDTA solution and fetal bovine serum (FBS, Gibco, USA) were purchased from Sigma-Aldrich. All chemicals were analytical grade and used without further treatment. The mPEG-PBLA was synthesized according to the literature (41) and dithiodipropionic anhydride was also synthesized according to previous report (42).

### Methods

^1^H NMR spectra were recorded in CDCl_3_ or DMSO-d_6_ solution on a Bruker (Switzerland) AVANCE III HD 400 MHz apparatus with the TSM proton signal for reference. IR measurements were performed using a Bruker VERTEX 70 model FT-IR. Dynamic light scattering experiments were performed with Nano-ZS90 Zeta-sizer from Malvern (Malvern Instruments, Malvern Co., UK) and morphology of the nanoparticles was investigated by Transmission electron microscopy (TEM) with JEM-2100 (Japan Electronics Co., Ltd.). The critical micelle concentrations were examined by Fluorescent spectrophotometer (Hitachi F-4600, Japan) at an excitation wavelength of 339 nm and emission wavelengths of 373 and 383 nm. *In vitro* release experiment was performed with a high-performance liquid chromatography system (HPLC, Waters 2695, Waters Corporation, Milford, USA) equipped with a Waters Symmetry C18 column (5 μm, 250 × 4.6 mm).

### Synthesis of PPSP and PPCP

PPSP was synthesized via two steps: an amidation and an esterification reaction. First, mPEG-PBLA was reacted with DTDPA to obtain mPEG-PBLA-SS-COOH. In detail, a solution of mPEG-PBLA (1.033 g) and 0.025 g DMAP (0.21 mmol) in 40 mL anhydrous dimethylformamide (DMF) with 0.20 mL of triethylamine (TEA) was added dropwise into a solution of DTDPA (0.23 g, 1.20 mmol) in 40 mL of anhydrous DMF at room temperature. The reaction was left to proceed for 24 h at 35°C under N_2_. The reaction mixture was concentrated in a vacuum to remove the DMF. The residue was partitioned between CH_2_Cl_2_ (DCM) (100 mL) and 0.5 mol/L aq. HCl (50 mL). The separated organic layer was washed with saturated brine (30 mL × 2), dried over MgSO_4_ and evaporated to dryness. Cold CH_3_CH_2_OH (10 mL) was added to the residue to form mPEG-PBLA-SS-COOH as a brown solid. Then, mPEG-PBLA-SS- COOH was reacted with PTX to obtain PPSP. Typically, 1.05 g mPEG-PBLA-SS- COOH, and 0.035 g DMAP (0.28 mmol) were dissolved in 50 mL of anhydrous CH_2_Cl_2_ (DCM), and the reaction mixture was stirred at 25°C for 1 h. Then, 0.128 g DCC (0.62 mmol) and 0.213 g PTX (0.25 mmol) were added successively, and the reaction was maintained at room temperature for another 48 h. Then, the suspension was filtered to remove the *N, N-*dicyclohexylurea (DCU), and the filtrate was washed with 0.5 mol/L aq. HCl (10 mL × 2) and saturated brine (10 mL × 2). The separated organic layer was dried over MgSO_4_ and evaporated to dryness. The products were first dialyzed against DMF (MWCO 5000) for 24 h, and then against H_2_O for another 24 h to remove unreacted impurities. The mPEG-PBLA-SS-PTX (PPSP) prodrug was collected and freeze-dried. As a control, uncleavable PPCP was prepared in a similar manner except DTDPA was replaced with succinic anhydride.

### Critical Micelle Concentration (CMC) of PPSP and PPCP

The critical micelle concentrations (CMCs) of PPSP and PPCP were determined via fluorescence spectrometry using pyrene as a fluorescent probe. Briefly, pyrene solution was prepared in acetone at a concentration of 5.0 × 10^−1^ g/L. Subsequently, aliquots of pyrene solution (0.5 mL) were added to test tubes, and the acetone was allowed to evaporate at ambient temperature. After that, 10 mL of the polymer prodrug solutions at concentrations ranging from 5.0 × 10^−6^ to 1.0 × 10^−1^ g/L was added to each tube and equilibrated for 24 h at room temperature. Finally, the samples were examined in a microplate reader (Hitachi F-4600, Japan) at an excitation wavelength of 339 nm and emission wavelengths of 373 and 383 nm. The fluorescence intensity ratio of the third and first vibronic bands (I_3_/I_1_) was plotted against the logarithm of concentration to obtain the CMC values.

### Preparation of Prodrug and Blank Micelles

The micelles were manufactured by a dialysis method. Firstly, 4 mg PPSP was resolved in 2 mL of DMSO at room temperature. The obtained solution was slowly dropped into 2 mL of water for injection under stirring at 1,600 rpm using a magnetic stirrer (Gongyi City Yuhua Instrument Co., Ltd., Gongyi, Henan, China). The mixture was transferred to dialysis tubes with a molecular weight cut-off of 3,500 Da after PPSP solution was added completely. The dialysis tube was placed in water for injection and incubated for 24 h. The water for injection was replaced by the refresh that every 2 h. Then, the dialysate was centrifuged by a table-type low-speed centrifuge at 4,310 g for 10 min (TGL-16B, Shanghai Anting Scientific Instrument Factory, Shanghai, China). The supernate was isolated to obtain the PPSP micelle.

PPCP and blank micelles were prepared by the same method as described above, except that PPSP was replaced by PPCP and mPEG-PBLA.

### Particle Size and Zeta Potential

Particle size, polydispersity index (PDI), and zeta potential were analysized by dynamic light scattering using a Zetasizer Nano-ZS90. Each sample was measured in triplicate, and the averages were calculated.

### Transmission Electron Microscopy

The dimensions and morphologies of the nanomicelles were observed at 80 kV by a JEOL 2010 transmission electron microscopy. Freshly prepared samples were placed onto a carbon-coated copper grid and air-dried at room temperature.

### Stability Study

#### Storage Stability

PPSP and PPCP were stored at 4°C in a refrigerator and at room temperature for 2 weeks. Approximately 2 mL of the samples was collected for analysis at 0, 1, 2, 4, 7, and 15 days, and the particle sizes were measured. All the experiments were repeated for three times.

#### Dilution Stability

To study the effect of dilution on the particle size stability, PPSP and PPCP were diluted 10, 50, and 100 times with water for injection, and the particle sizes of the samples were measured using a Malvern Zetasizer as described above. All the experiments were repeated for three times.

### Redox-Responsive Behavior of PPSP and PPCP

To investigate the redox-responsive behavior of PPSP (1.5 mL), changes in the micelle size in response to different GSH levels (0, 2 μM, 10, and 40 mM) were detected via DLS on an orbital shaker in an incubator at 37°C with horizontal agitation (100 rpm). The particle size change of the micelles after incubation with different GSH levels for 24 h was analyzed by DLS. At the same time, the redox-responsive behavior of PPCP was also assessed as a contrast with that of PPSP under the same conditions. Three copies of each test sample were analyzed in parallel.

### *In vitro* Release Study

To assess drug release from the micelles, 2 mL of PPSP was poured into dialysis tubes (MWCO 2000), which were placed in 10 mL of 0.01 M PBS (pH 7.4, 0.5% w/v Tween-80) with different GSH levels (2 μM, 10, and 40 mM). The samples were incubated on an orbital shaker in an incubator at 37°C with horizontal agitation (150 rpm). To monitor release, 1 mL of medium was withdrawn and replaced with an equal volume of fresh medium at specific time points: 0.5, 1, 2, 4, 6, 12, 24, 36, and 48 h. The total amount of released PTX was determined by HPLC. HPLC analyses were performed with a high-performance liquid chromatography system (HPLC, Waters 2695, Waters Corporation, Milford, USA) equipped with a Waters Symmetry C18 column (5 μm, 250 × 4.6 mm). The mobile phase consisted of acetonitrile/water (3: 2 v/v) at a flow rate of 1.0 mL/min. The column temperature was maintained at 35°C. The detection wavelength was 227 nm, and the sample injection volume was 20 μL. A standard curve was drawn using a PTX reference solution at a concentration range of 0.5–20.0 mm g/mL.

### *In vitro* Cell Assay

The cytotoxicity of PTX, PPSP, and PPCP against HepG2, MCF-7, and HL-7702 cells was evaluated using MTT assays. HepG2, MCF-7, and HL-7702 cells were cultured in DMEM supplemented with 10% fetal bovine serum (5% CO_2_ at 37°C). The cells were seeded into 96-well plates at a density of 6 × 10^3^ cells/well and incubated for 24 h to allow cell attachment. Next, the cells were treated with various concentrations of PTX, PPSP, PPCP, and blank micelles (mPEG-PBLA, corresponding to the maximum concentration of the formulation). After incubation for 24 and 48 h, 20 μL of MTT (5 mg/mL) solution was added to each well. The plate was incubated for an additional 4 h to allow viable cells to reduce the yellow tetrazolium salt (MTT) into purple-blue formazan crystals, and then, the medium was removed. DMSO (200 μL) was added to each well to dissolve any purple formazan crystals formed. The plates were vigorously shaken before measurement of relative color intensity. The absorbance of each well was measured at a wavelength of 570 nm with a plate reader. The results are expressed as the mean values of three measurements.

### Statistical Analysis

All the experiments were repeated three times, the statistical significance of differences between groups was conducted using the model independent (similarity factor) method.

## Results

### Preparation and Characterization of the PPSP and PPCP Prodrugs

**Figure 1:**
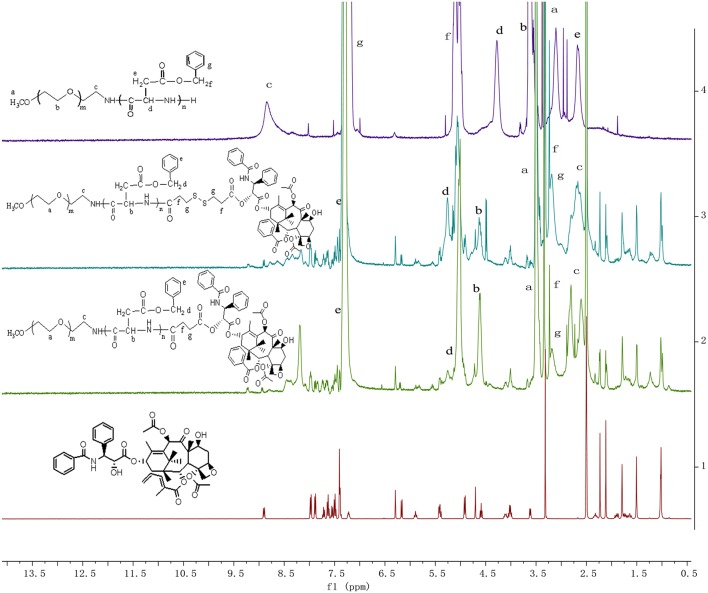
The ^1^H NMR spectra of PPCP, PPSP, mPEG-PBLA, and PTX in DMSO-d6.

The synthesis route for the PPSP prodrug is shown in Figure S1. DCC and DMAP were used to catalyze the esterification reaction of PTX with the disulfide linker to obtain the PPSP prodrug. The chemical structure of PPSP and PPCP was determined by ^1^H NMR (Figure 1) and FT-IR spectroscopy. The presence of PEG-PBLA was confirmed by the proton peaks at δ = 4.63 ppm (-COCH-), δ = 2.66 ppm (-CH_2_COO-), δ = 5.05 ppm (OCH_2_-C_6_H_5_), and δ = 7.31 ppm (-Ar-H). The strong proton peaks of -CH_2_CH_2_O- are shown at δ = 3.51 ppm, indicating that the PEG chain was grafted to the PBLA chain. Compared with the spectra of PXT, mPEG-PBLA-CC-COOH and mPEG-PBLA-SS-COOH (Figure S2), the peaks of -COOH at δ = 12.14 ppm and δ = 12.36 ppm disappeared and the new proton peaks of PTX appeared in the PPCP and PPSP spectra, which indicated the PTX was successfully grafted to the -COOH groups of mPEG-PBLA-CC-COOH and the mPEG-PBLA-SS-COOH. In summary, the ^1^H NMR spectra suggest that the PPCP and PPSP prodrugs were successfully synthesized.

FT-IR spectra provided clear evidence for the successful preparation of the PPSP and PPCP conjugates (Figure S3). The spectrum of the standard PTX shows characteristic peaks at 1,734, 1,714 cm^−1^ which are assigned to the symmetric stretch of C = O. The peaks at 1,646 cm^−1^ is assigned to the stretch of C = O, and the peaks at 3,300–3,500 cm^−1^ is assigned to the extractable vibration peak of O-H. It was illustrated in FT-IR spectra that mPEG-PBLA had stretching vibration band of CH_3_O-(CH_2_-CH_2_O)n in 2,887 cm^−1^, C = O of carboxyl group in 1,740 cm^−1^, benzene ring in 1,658 cm^−1^ (Amide I), and 1,554 cm^−1^ (Amide II), CH_3_O-(CH_2_-CH_2_O)_n_ in 1,110 cm^−1^. As for PPSP, a new peak related to the C-H bent vibration of -CH_2_-S-S-CH_2_- appeared at 1,413 cm^−1^. Compare to PPCP, peaks at 1,200 cm^−1^ is assigned to the asymmetric stretch of single C = O which was characteristic peaks related to the polyacrylate.

**Figure 2:**
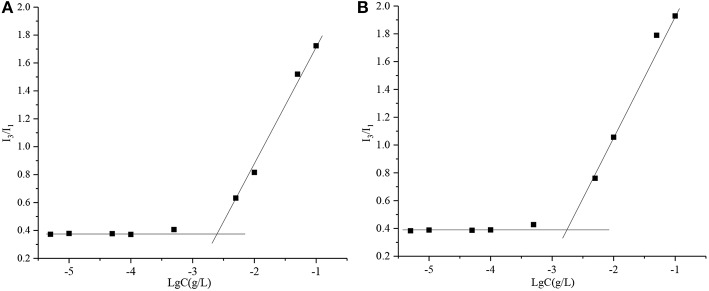
The CMC of PPSP **(A)** and PPCP **(B)**.

Critical Micelle Concentration (CMC) is the key parameter that represents the self-assembly performance of an amphiphilic polymer. A small CMC is an essential condition to prepare stable micelles. In this study, the CMCs of PPSP and PPCP were assessed. The CMCs of PPSP and PPCP were calculated as 2.97 μg/mL (Figure 2A) and 2.10 μg/mL (Figure 2B) according to the data shown in Figure 2 and thus were similar. This implies that PPSP and PPCP would have similar stability. The low CMC ensures the self-assembly behavior of PPSP and PPCP, which indicates good stability of the two micelles after injection into the blood circulation (31, 42).

### Particle Size, Zeta Potential, and Morphology

The particle size and zeta potential of PPSP and PPCP were analyzed using dynamic light scattering technology. The results are presented in Table 1 and Figure S4. The results show that the prepared PPSP and PPCP have a very similar particle size, without significant differences among different batches. The dispersion coefficient (PDI) of PPSP and PPCP was small, indicating good repeatability of the applied preparation and uniform dimension of particle size in the prepared micelles. The particle sizes of PPSP and PPCP were 63.77 ± 0.91 and 61.24 ± 0.81 nm, respectively. This small particle size is very beneficial for targeted accumulation of polymer micelles in tumor tissues. Nanoparticles in a size range of 10–200 nm are difficult to eliminate through the kidneys or mononuclear macrophages from blood circulation, but they can leak out from blood vessels and enter into tumor spaces through the EPR effect (43, 44). The zeta potentials of PPSP and PPCP were −25.8 ± 3.24 and −31.57 ± 2.22 mv, respectively. These values indicate that both PPSP and PPCP load more static charges. Thus, a strong mutual repulsive force is present among colloidal particles, which is conducive to the stability of the micelle solution (41) Negative charge is also beneficial for long micelle circulation and biosafety and the accumulation of micelles in tumors through the EPR effect (45).

**Table 1:** Reproducibility study of the micellar prescription.

**Sample**	**Particle size (nm)**	**PDI**	**Zeta (mV)**
PPSP	63.77 ± 0.91	0.162 ± 0.023	−25.8 ± 3.2
PPCP	61.24 ± 0.81	0.079 ± 0.036	−31.6 ± 2.2

**Figure 3:**
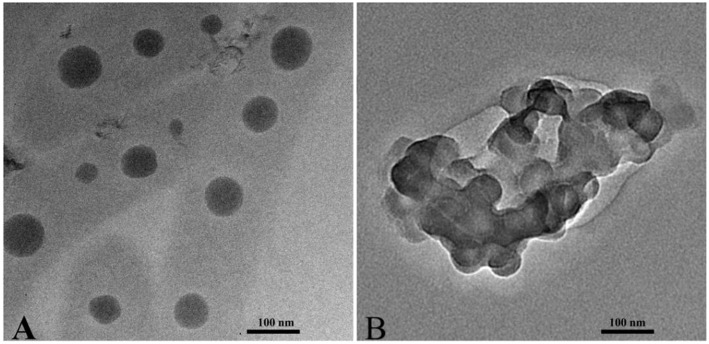
TEM images of micelles formed by PPCP **(A)** and PPSP **(B)**.

Transmission electron microscopy (TEM) imaging results show that PPSP and PPCP are spheres with a uniform dimension (Figure 3). The grain sizes of PPCP were ~40 (Figure 3A) and 45 nm (Figure 3B), smaller than the sizes measured with dynamic light scattering (~62 nm, Table 1). This difference might be attributed to the different particle size measurement technologies. The hydrophilic PEG shell of the prepared PPSP and PPCP micelles shrinks via dehydration during sample drying when preparing samples for TEM, and thus, the PEG is not fully extended (31).

### Stability Study

**Figure 4:**
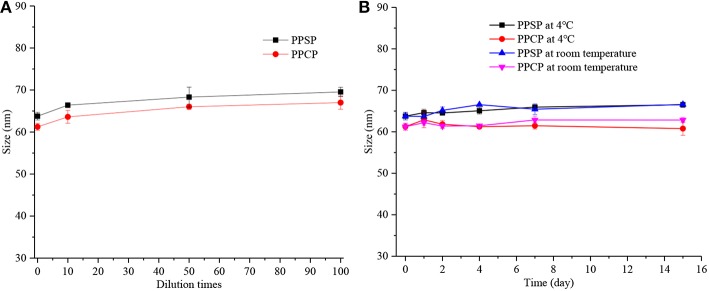
The dilution stability **(A)** and storage stability **(B)** assay results.

Good stability is essential for application and development of a nano-drug delivery system. High dilution stability is very important for nanoparticles intravenously injected into blood circulation. In addition, physical stability during storage is vital to druggability and the effectiveness of nanoparticles. Therefore, the dilution stability and storage stability of PPSP and PPCP were investigated in this study (Figure 4). It can be seen from Figure 4A that the particle size of both PPSP and PPCP changed within 10 nm after they were diluted by 10, 50, and 100-fold, confirming their good dilution stability. These results indicate that PPSP and PPCP would maintain good stability in blood circulation (37, 41). Figure 3B shows that the dimension of PPSP and PPCP fluctuates within 5 nm after storage at 4°C and at room temperature for 15 days. The particle size changed slightly more after storage at room temperature for 15 days than at 4°C. This reflects the good physical stability of PPSP and PPCP, thus assuring long-term stability (37, 41). On the one hand, the good stability of PPSP and PPCP is attributed to the small CMC of the polymer prodrugs, which produces a satisfying self-assembly behavior. On the other hand, a π-π conjugation effect may occur between PTX molecules in the hydrophilic segment of the polymer prodrugs, which can increase auto-agglutination of the hydrophilic segment and thereby strengthen core agglomeration in micelles (32, 46, 47).

### Redox-Responsive Behavior of PPSP and PPCP

**Figure 5:**
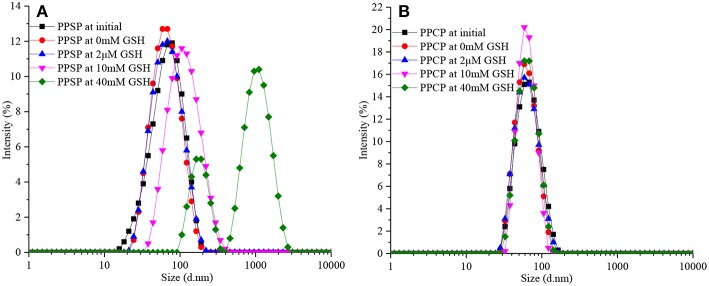
Particle sizes change of PPSP **(A)** and PPCP **(B)** micelles at different GSH concentrations.

The redox-responsive self-assembly behavior and drug release of polymer prodrugs can be predicted by measuring changes in particle size in environments with different level of reductive. The influence of GSH concentration on PPCP and PPSP particle size was analyzed in this study (Figure 5). In Figure 5A, the particle size and PDI of PPSP remained basically the same when the GSH concentration changed between 0 and 2 μM (GSH concentration in human blood), which indicates that the micelles can maintain stable aggregation in human blood circulation. The particle size of PPSP began to increase when the GSH concentration reached 10 mM, followed by a sharper increase under the higher GSH concentration (40 mM). The distribution curve of PPSP particle size changed from one to two peaks. This result reveals that the disulfide bond in PPSP molecules contributes to their strong reduction sensitivity, allowing PPSP to specifically release drug in tumor cells (48–50). The change in particle size distribution of PPSP from one peak to two peaks can be interpreted as follows. Disulfide bonds in PPSP molecules break quickly in an environment with a high GSH concentration, which induces the release of a large amount of PTX and disappearance of π-π conjugation between PTX molecules in the micelle cores (32, 46, 47). As a result, the agglomeration force in the hydrophilic segment is weakened, and the micelle immediately collapses. The newly generated mPEG-PBLA rearranges irregularly into polydisperse second-order micelle particles (37). In contrast, the particle size and PDI of PPCP changed only slightly after incubation with different concentrations (0 mM, 2 μM, 10 mM, and 40 mM) of GSH solution within 24 h (Figure 5B), demonstrating that PPCP was not redox sensitive. This is likely because the PPCP molecules do not contain disulfide bonds, and the C-C bond that connects mPEG-PBLA and PTX has no redox sensitivity (46, 47).

### *In vitro* Release Study

**Figure 6:**
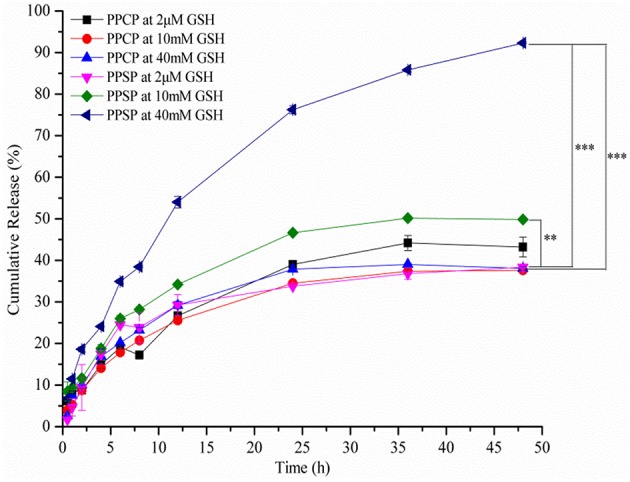
Redox-sensitive release of drug from PPSP and PPCP micelles.

Studies on *in vitro* release of drugs can simulate the *in vivo* release behavior of preparations. In this study, the influence of different reductive environment on drug release from polymer micelles *in vitro* was investigated with PPCP as a control. The cumulative release curve of PPSP was tested in environments with different reduction levels (Figure 6). It can be seen from Figure 6 that PPSP released drugs slowly in an environment with 2 μM GSH (simulating the human blood environment). The released PTX amount at 48 h was only 38.6%. However, the drug release of PPSP was significantly accelerated when the GSH concentration was increased to 10 and 40 mM (simulating the environment in tumor cells), and the released PTX content at 48 h reached 49.5 and 92.5%, respectively. When GSH concentration was increased to 40 mM, comparable to reported intracellular GSH level in tumor cells (17, 42, 51). The drug release of PPCP was slow under all concentrations of GSH solution (Figure 6). The cumulative PTX content released by PPCP within 48 h under three GSH concentrations (2 μM, 10 mM, and 40 mM) was 44.9, 37.4, and 38.3%, respectively, without significant differences. In sum, PPSP with high reduction sensitivity can release drugs in quick response to a high GSH concentration, whereas PPCP without reduction sensitivity basically maintains the same release speed regardless of changes in GSH concentration. This conclusion is consistent with the experimental results showing the responses of micelle grain size to a reducing environment (section “Redox-Responsive Behavior of PPSP and PPCP”). Hence, PPSP remains stable in blood circulation and can prevent premature release of drug cargo. Nevertheless, once PPSP reaches the tumor tissue through the EPR effect and is swallowed by tumor cells, it can rapidly release drugs in tumor cells with high GSH concentration and achieve specific accumulation. Such release behavior is not observed with PPCP. It can be speculated that intravenous injection of PPSP can accumulate drugs into tumor cells more effectively and thereby achieve a better anti-tumor effect than PPCP.

### *In vitro* Cell Assay

**Figure 7:**
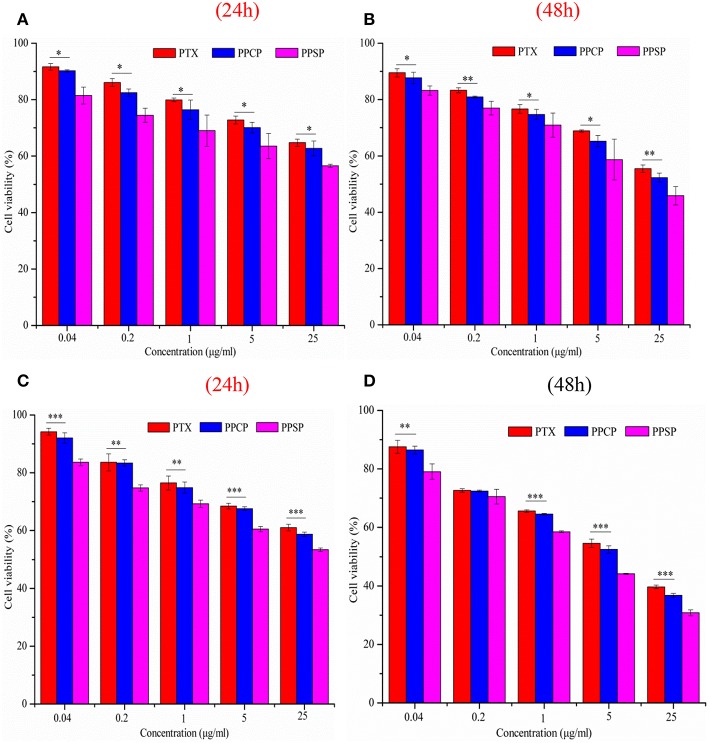
Cytotoxicity of micelles against HepG2 cells **(A,B)** and MCF-7 cells **(C,D)** for 24 and 48 h, ^*^P < 0.05, ^**^P < 0.01, ^***^P < 0.001.

The inhibitory effect of blank micelles on *in vitro* growth of HepG2 and MCF-7 cells at different stages was investigated first to evaluate the biosafety of the polymer carrier. The results are shown in Figure S5. The inhibition rate of the polymer carrier PEG-PBLA in HepG2 cells and MCF-7 cells was <10% at 24 and 48 h. These results not only confirm the non-toxicity of PEG-PBLA to HepG2 and MCF-7 cells but also suggest good biosafety of PEG-PBLA. This conclusion is in accordance with our previous acute toxicity experimental results (41). In this study, the *in vitro* toxicity of different concentrations of PPSP toward HepG2 cells. MCF-7 cells and non-cancerous HL-7702 cells was tested with free PTX and PPCP as the controls. The experimental results showing the toxicity of the three PTX preparations in HepG2, MCF-7 cells, and HL-7702 cells are shown in Figure 7 and Figure S6. The survival rate of HepG2 cells (Figures 7A,B) and MCF-7 (Figures 7C,D) cells declined gradually with an increase in drug concentration. In addition, the survival rate of HepG2 and MCF-7 cells decreased as time elapsed (from 24 to 48 h) under the same drug concentration. In a word, the inhibitory effect of all three PTX preparations on the growth of HepG2 and MCF-7 cells was related to the PTX concentration and time. Meanwhile, from Figure S6 we can see that PPSP has low toxicity against non-cancerous HL-7702 cells compared with PTX and PPCP. Furthermore, Table 2 shows that the IC_50_ for PPSP inhibition of HepG2 and MCF-7 cell growth was significantly lower than that of free PTX and PPCP. The IC_50_ of free PTX was the highest, while the IC_50_ of PPCP was slightly lower than that of free PTX. At 24 and 48 h, the IC_50_ of PPSP was ~8.6–18.2% of the IC_50_ of PPCP and 2.9–11.1% of the IC_50_ of free PTX. All these results confirm the concentration and time dependence of toxicity of the three PTX preparations against HepG2 and MCF-7 cells. Compared with free PTX, PPSP, and PPCP inhibited the growth of HepG2 and MCF-7 cells more potently. This phenomenon might result from the ability of polymer prodrug micelles to facilitate drug uptake by tumor cells through endocytosis digestion and to carry the drug into the cytoplasm while allowing escape from lysosomes. PPSP can release drug quickly upon stimulation with a high GSH concentration in the cytoplasm of tumor cells to quickly increase the concentration of free drug over the treatment concentration, thus effectively and quickly realizing the goal of killing tumor cells. Although PPCP can increase drug intake by tumor cells, it cannot release drugs quickly in the cytoplasm without a response to GSH. Therefore, the intracellular concentration of free drug is too low to kill tumor cells. Free PTX can only enter into cells through a passive diffusion mechanism, which leads to a low drug concentration in cells and weak anti-tumor activity.

**Table 2:** IC_50_ values (μg/mL) of PPSP, PPCP, and free PTX against MCF-7 and HepG2 cells after treatment for 24 and 48 h.

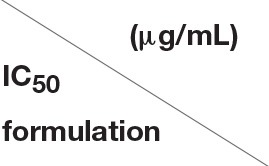	**MCF-7**		**HepG2**	
	**24 h**	**48 h**	**24 h**	**48 h**
PTX	42.4	1.8	5,717.9	891.3
PPCP	24.6	1.1	1,942.3	713.5
PPSP	3.7	0.2	166.3	27.9
Ratio^a^ (%)	8.8	11.1	2.9	3.1
Ratio^b^ (%)	15.0	18.2	8.6	3.9

a *Percentage of PPSP micelle to free PTX*,

b *Percentage of PPSP micelle to PPCP*.

## Conclusions

In this study, PPSP and PPCP were first synthesized and then assembled into a polymer prodrug micelle through dialysis. The average particle sizes of PPSP and PPCP were 63.77 ± 0.91 and 61.24 ± 0.81 nm. The PDI of PPSP and PPCP was <0.2, and their zeta potential was higher than −25 mV. In stability experiments, both PPSP and PPCP presented good dilution stability and storage stability, which lay the foundation for intravenous injection and long-term storage. Redox-responsive and *in vitro* release experiments confirmed the high redox-responsive sensitivity of PPSP. The particle size of PPSP changed significantly under high GSH concentration conditions, and PPSP rapidly released PTX, allowing the drug to accumulate in tumor cells. Moreover, the inhibitory effect of PPSP against HepG2 and MCF-7 cells was investigated, and the results revealed that PPSP inhibited the growth of HepG2 and MCF-7 cells more potently than PPCP and free PTX. These results indicate that PPSP can increase the anti-tumor effect of PTX and possesses promising application prospects for the study of new PTX preparations. However, further studies on the mechanisms by which PPSP improves drug intake by tumor cells, and the *in vivo* tumor activity and tissue distribution are still needed.

## Data Availability

All datasets generated for this study are included in the manuscript/Supplementary Files.

## Author Contributions

SC and TZ were mainly involved in the synthesis of prodrug materials. SC was in charge of the synthesis work. YW was mainly responsible for the specific experimental work including the preparation and evaluation of the micelles. LZo, HZ, and LZh participated in part of the experiment. XP designed the research project in general, mainly responsible for the evaluation of the micelles, and gave specific instructions in the specific experiment. BF gave financial support to this project and gave some valuable suggestions for revision of the article.

### Conflict of Interest Statement

The authors declare that the research was conducted in the absence of any commercial or financial relationships that could be construed as a potential conflict of interest.
