# Supplementary material for: Redox-Responsive Disulfide Bond-Bridged mPEG-PBLA Prodrug Micelles for Enhanced Paclitaxel Biosafety and Antitumor Efficacy
Source: Front Oncol. 2019 Aug 27;9:823. doi: 10.3389/fonc.2019.00823 (PMC6719549; doi:10.3389/fonc.2019.00823)
Supplement: Supplementary file 1 [file Data_Sheet_1.docx]

**Redox-responsive disulfide bond-bridged mPEG-PBLA prodrug micelles for enhanced paclitaxel biosafety and antitumor efficacy**

Sheng Chang^1*#^, Yanfei Wang^2#^, Tianyi Zhang^1^, Xiaohui Pu^2*^, Lanlan Zong^2^, Heyun Zhu^1^, Luling Zhao^2^, Bo Feng^1*^

^1^College of Pharmacy, Jilin Medical University, Jilin, Jilin, 132013, China

^2^Institute of Materia Medica, School of Pharmacy, Henan University, Jinming Road, Kaifeng Henan, 475004, China

***#*** *These authors contributed equally.*

Correspondence:

Xiaohui Pu

Institute of Materia Medica, School of Pharmacy, Henan University, Jinming Road, Kaifeng Henan, 475004, China

Tel./fax: +86 371 2388 0680

E-mail: [pgh425@163.com](mailto:pgh425@163.com)

Sheng Chang

College of Pharmacy, Jilin Medical University, Jilin, Jilin, 132013, China

Tel./fax: +86 432 6456 0532

E-mail: [changsheng-pharm@hotmail.com](mailto:changsheng-pharm@hotmail.com)

Bo Feng

College of Pharmacy, Jilin Medical University, Jilin, Jilin, 132013, China

fengbo2@sina.com

Fig. S1 The synthesis route of the polymer prodrug

Fig. S2 The ^1^H NMR spectra of mPEG-PBLA-CC-COOH、mPEG-PBLA-SS-COOH in DMSO-d6

Fig. S3 FT-IR spectra of the polymer prodrug

Fig. S4 The particle sizes of prodrug micelles





Fig. S5 Cytotoxicity of blank micelles against HepG2 and MCF-7 cells at different stages

Fig. S6 Cytotoxicity of different formulations against 7702 cells at 24h
